# Nanoscale plasma-activated aerosol generation for in situ surface pathogen disinfection

**DOI:** 10.1038/s41378-022-00373-3

**Published:** 2022-04-14

**Authors:** Nicholas S. L. Chew, Kiing S. Wong, Wei S. Chang, Chien W. Ooi, Leslie Y. Yeo, Ming K. Tan

**Affiliations:** 1 Mechanical Engineering Discipline, School of Engineering, Monash University Malaysia, Bandar Sunway, Selangor Malaysia; 2 Chemical Engineering Discipline, School of Engineering, Monash University Malaysia, Bandar Sunway, Selangor Malaysia; 3 Micro/Nanophysics Research Laboratory, RMIT University, Melbourne, VIC Australia

**Keywords:** Microfluidics, Nanostructures

## Abstract

Plasma treatment constitutes an efficient method for chemical-free disinfection. A spray-based system for dispensing plasma-activated aerosols onto surfaces would facilitate disinfection of complex and/or hidden surfaces inaccessible to direct line-of-sight (for example, UV) methods. The complexity and size of current plasma generators (for example, plasma jet and cometary plasma systems)—which prohibit portable operation, together with the short plasma lifetimes, necessitate a miniaturized in situ technique in which a source can be simultaneously activated and administered on-demand onto surfaces. Here, we demonstrate this possibility by combining two nanoscale technologies for plasma and aerosol generation into an integrated device that is sufficiently small and lightweight. Plasma is generated on a carpet of zinc oxide nanorods comprising a nanoneedle ensemble, which when raised to a high electric potential, constitutes a massive point charge array with near-singular electric fields to effect atmospheric breakdown. The plasma is then used to activate water transported through an underlying capillary wick, that is subsequently aerosolized under MHz-order surface acoustic waves. We show that the system, besides being amenable to miniaturization and hence integration into a chipscale device, leads to a considerable improvement in plasma-activation over its macroscale cometary discharge predecessor, with up to 20% and 127% higher hydrogen peroxide and nitrite ion concentrations that are respectively generated in the plasma-activated aerosols. This, in turn, leads to a 67% reduction in the disinfection time to achieve 95% bacterial load reduction, therefore demonstrating the potential of the technology as an efficient portable platform for on-demand field-use surface disinfection.

## Introduction

Conventional disinfection and sterilization techniques, which includes the use of chemical sanitizers and ultraviolet (UV) disinfection, are often restricted by their inability to effectively inactivate a sizable percentage of bacteria and viruses, among other drawbacks^1–3^. Chemical sanitizers, for example, can sometimes pose safety issues for consumers as they often leave residues that could deteriorate an already contaminated surface^4,5^. Ultraviolet (UV) disinfection, on the other hand, requires longer treatment times and direct line-of-sight exposure and is hence ineffective for decontaminating areas shielded by objects. Additionally, UV irradiation can also, at times, lead to serious infections due to the photo-reactivation of pathogenic bacterium^3^. In recent years, studies have shown plasma-activated water as a promising alternative for surface disinfection^6–8^.

Plasma comprises an ionized gas that consists of electrons, positive and negative ions, neutral and excited atoms, ground and excited state molecules, and UV photons, along with free radicals^9^. When plasma comes into contact with water, reactive species are generated at the liquid–gas interface and diffuse into the liquid, generating plasma-activated water that contains reactive species; the presence of these reactive species leads to lower pH values and higher electric conductivity of the sample. More importantly, these reactive species, which consist of reactive oxygen species and reactive nitrogen species, such as nitric oxide radicals, nitrite, nitrate, atomic oxygen and ozone, in plasma-activated water, can give rise to antimicrobial effects^10,11^ by inducing high oxidative stresses on bacteria and other pathogens.

There are many common ways to generate plasma to treat water, including the use of corona discharge, dielectric barrier discharge and atmospheric pressure plasma jets, each possessing their own advantages. Corona discharge is a simple and inexpensive technique while dielectric barrier discharge provides the ability to minimize adverse degradation (for example, etching and corrosion) of the electrodes since they are separated by a dielectric barrier. Atmospheric pressure plasma jets, on the other hand, provide a stable and strong plasma with easily controllable gas discharge temperature. These techniques are often employed for the bulk production of plasma-activated water, which often requires treatment times in excess of 15 min, and usually several hours^7,8^. However, the storage of bulk quantities of plasma-activated water can be challenging. A previous study has shown that the sterilization efficiency of plasma-activated samples typically degrade over time, the rate of which is affected by the storage temperature^12^. The half-life of reactive species in their plasma-treated water was shown to be 21 days, 2.9 days, 74 min, 4.33 min and 0.8 min when stored at − 30 ^∘^C, − 18 ^∘^C, 0 ^∘^C, 15 ^∘^C, and 25 ^∘^C, respectively^13^. Another study also showed that if plasma-treated water was stored immediately at − 80 ^∘^C following plasma treatment, it was possible to maintain the degradation rate of hydrogen peroxide at 20%, in addition to prolonging the degradation of nitrite ions over 30 days^14^. Such storage requirements are, however, impractical in the majority of practical cases.

As such, a rapid and miniaturized system that facilitates on-demand generation of plasma-activated water at the point-of-use for surface decontamination constitutes a significant practical advantage over conventional systems. While our previous study conceptually demonstrated the possibility of generating aerosols with a portable nebulization system that was subsequently plasma-activated using cometary discharge for spray-deposition of surfaces for bacterial inactivation^15^, the use of two cometary discharge systems to provide sufficient uniform areal plasma coverage to activate the water feed to the nebulizer to be aerosolized, however, prevented integration into a compact and lightweight miniaturized setup that facilitates portable operation.

In this work, we utilize the potential of both nanoscale plasma and aerosol generation systems in tandem to demonstrate an efficient and portable system for in situ production of plasma-activated aerosols that can be used for surface pathogen decontamination on-demand. The former comprises zinc oxide (ZnO) nanorods that resemble thousands of nanoscale needles with near-singular electric fields, which we show, for the first time, can be efficiently charged to produce atmospheric pressure plasma with a high concentration of reactive species over a large coverage area, without the need for multiple plasma generators^15^. The latter, on the other hand, involves the use of chip-scale high frequency nanometer amplitude electromechanical excitation in the form of surface acoustic waves (SAWs), whose unprecedented surface vibrational acceleration on the order of 10 million *g*’s provides a mechanism for interfacial destabilization^16^ that is highly effective for the generation of micron-dimension aerosol droplets^17,18^ for applications across pulmonary drug delivery^19^, thermal management^20^, water filtration^21^, thin-film deposition^22^, mass spectrometry^23,24^, and material synthesis/crystallization^25,26^. Additionally, SAWs have been shown to be a powerful vehicle for manipulating microscale fluids for a host of microfluidic applications, such as liquid and drop transport^27,28^, tensiometry^29^, mixing^30,31^, sorting^32^, heating^33^, greywater treatment^34^, and bubble/particle trapping and manipulation^35–38^.

## Results and discussion

**Fig. 1:**
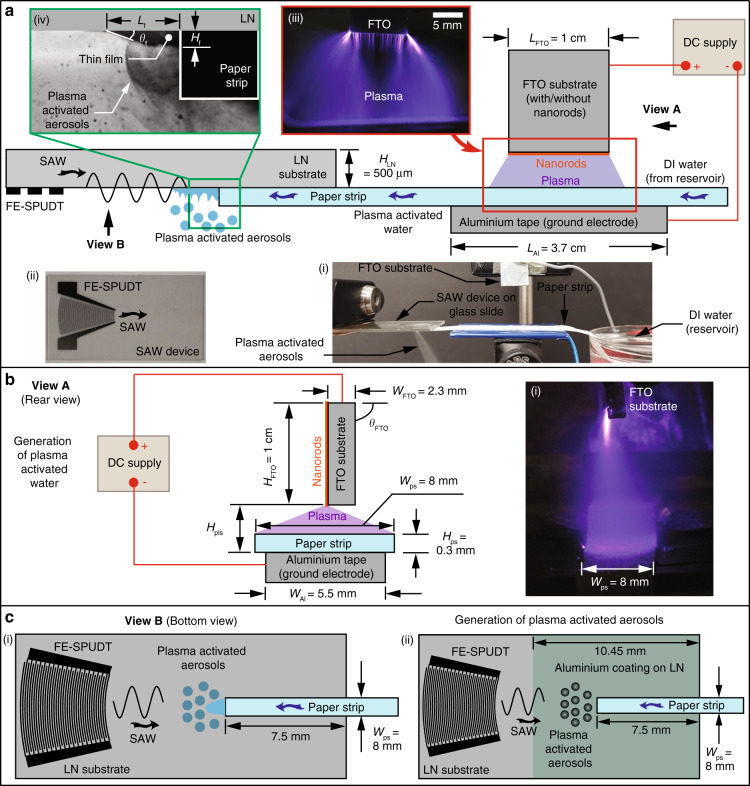
The experimental setup comprises an atmospheric pressure plasma generator and a SAW nebulizer. **a** Side-view sketch (and image, as shown in the inset (**a**–i)) of the experimental setup, which comprises an atmospheric pressure plasma generator and a SAW nebulizer. The SAW device (as shown in inset (**a**–ii)), which comprises a chip-scale piezoelectric (lithium niobate, LN) substrate, is inverted to facilitate the deposition of the aerosols onto a test tube for subsequent characterization. Water from a reservoir is transported to the surface of the SAW device via a paper strip through both capillary and SAW-driven transport. As it travels through the paper strip, the water is exposed to the plasma generated by the atmospheric pressure plasma generator that consists of the fluorine-doped tin oxide (FTO) substrate on which ZnO nanorods are grown and a ground electrode (aluminium tape beneath the paper strip), both of which are connected to a high voltage DC supply. The inset (**a**–iii) shows a magnified image of the plasma generated between the nanorods and the ground electrode that is directed at the paper strip through which the water from the reservoir travels and from which a thin film emanates on the SAW device, where it is nebulized to generate plasma-activated aerosols, as shown in inset (**a**–iv). **b** Rear view (View A) sketch illustrating the plasma generation, whose dispersion is sufficiently wide to cover the entire width of the paper strip *W*_ps_, as shown in the inset (**b**–i). (**c**–i) Bottom view (View B) sketch illustrating the SAW device on which the thin film is nebulized to produce the plasma-activated aerosols. (**c**–ii) Sketch showing a 200 nm thick aluminium coating in the nebulization zone on the LN substrate surface that leads to enhancement of the nebulization rate by suppressing the electric field. All sketches are not to scale

The experimental setup is shown in Fig. 1, and consists of two major components: the chip-scale SAW nebulizer and the plasma generator comprising a fluorine-doped tin oxide (FTO) substrate on which ZnO nanorods are grown to generate atmospheric pressure plasma by applying a high voltage direct current (DC) across the nanorods and a grounded substrate. Upon excitation of the device, liquid is drawn by the SAW from a liquid reservoir through a paper strip onto the SAW chip, where it nebulizes to form the aerosols. While in transit through the paper strip, the liquid is exposed to the plasma generated by the ZnO nanorods above it to generate the plasma-activated water for subsequent aerosolization.

### Plasma generation

**Fig. 2:**
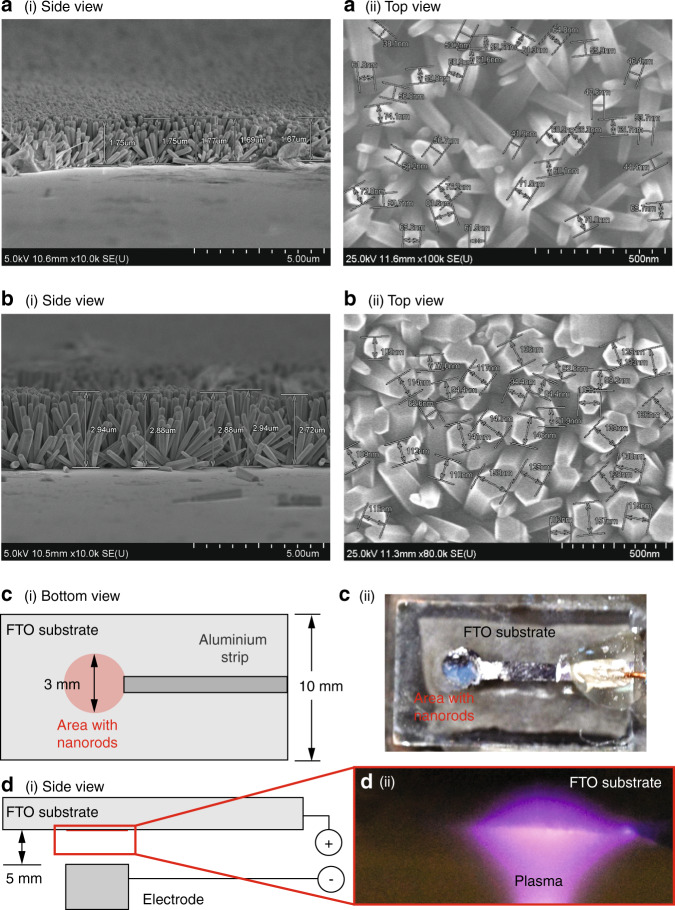
ZnO nanorods on the FTO substrates and the generation of atmospheric pressure plasma on the surface of ZnO nanorods. Side- and top-view field-emission scanning electron microscope (SEM) images of the hexagonally shaped ZnO nanorods grown on the FTO substrates after **a** 1 h and **b** 3 h of annealing. The length *L*_rod_ and diameter *ϕ*_rod_ of the nanorods were estimated from these images. **c**–i Bottom- and **d**–i side-view sketches (not to scale) showing the circular area (approximately 3 mm in diameter) where the aluminium-coated ZnO nanorods are grown on the 20 mm × 10 mm FTO substrate. A strip of aluminium coating with a width of ~1.5 mm links the circular area to the opposite edge of the FTO substrate to facilitate connection to the high voltage power supply. Also shown are **c**–ii a top-view image of the FTO substrate coated with the nanorods, and **d**–ii a side-view image showing the generation of atmospheric pressure plasma on the surface of nanorods after high voltage (≈6 kV) is applied across the nanorods and the ground electrode, which is separated by a gap of 5 mm from the nanorods.

The dimension of the ZnO nanorods that were grown on the FTO substrate can be controlled by different parameters such as the annealing duration, ZnO seed density, surfactant concentration and the concentration of the Zn starting material, among other parameters^39–42^. In this work, we varied the annealing duration *t*_An_ to obtain slightly different nanorod dimensions. Consistent with that reported elsewhere^43^, the mean length *L*_rod_ and diameter *ϕ*_rod_ of the hexagonally shaped ZnO nanorods can be seen from Fig. 2a, b to increase by approximately 75% and 83%, respectively, from *L*_rod_ = 1.6 ± 0.2 *μ*m and *ϕ*_rod_ = 60 ± 20 nm to *L*_rod_ = 2.8 ± 0.2 *μ*m and *ϕ*_rod_ = 110 ± 50 nm when *t*_An_ was extended from 1–3 h. Additionally, we also observed that the nanorods were more densely packed with longer annealing times.

Application of a high voltage (several kV) across the aluminium-coated ZnO nanorods and a ground electrode can be seen to result in plasma generation (Fig. 2d), which was notably absent with bare FTO substrates. For the production of plasma-activated water, ZnO nanorods were fabricated on one side of the FTP substrate, and it can be seen from Fig. 1(a–iii) and 1(b–i) that the entire paper strip, with a width of 8 mm and a length greater than 10 mm, is exposed to the plasma that is generated by the thousands of nanorods, each of which resembles a nanoscale needle. This is, in contrast, to the cometary discharge setup using a single (or two) point sources in preceding work^15^, where exposure across a small spot of approximately *ϕ*_pls_ ≈ 3 mm on the paper strip could only be achieved albeit at higher intensities.

### Plasma activation

To evaluate the effectiveness of the nanorods in concert with the SAW nebulizer in producing the plasma-activated aerosols, we examined six different configurations: (Configuration-1) bare FTO substrate, (Configuration-2) FTO substrate with a 200 nm aluminium layer, (Configuration-3) FTO substrates with *ϕ*_rod_ ≈ 60 nm ZnO nanorods, (Configuration-4) FTO substrates with *ϕ*_rod_ ≈ 60 nm ZnO nanorods coated with a 200 nm aluminium layer, (Configuration-5) FTO substrates with *ϕ*_rod_ ≈ 110 nm ZnO nanorods, and, (Configuration-6) FTO substrates with *ϕ*_rod_ ≈ 110 nm ZnO nanorods coated with a 200 nm aluminium layer. Configuration-1 was included given that the FTO substrate is electrically conductive^44^, especially relative to ZnO, which is a semiconductor^45^, and therefore there is a possibility of plasma generation, albeit at lower intensities, at the edges of the bare substrate. To increase the conductivity of the ZnO nanorods, a 200 nm aluminium layer was included in Configuration-4 and Configuration-6 to facilitate electron flow to the nanorods to facilitate sufficient charging at their tips so that the critical threshold voltage required for dielectric breakdown of the surrounding atmosphere is exceeded.

**Fig. 3:**
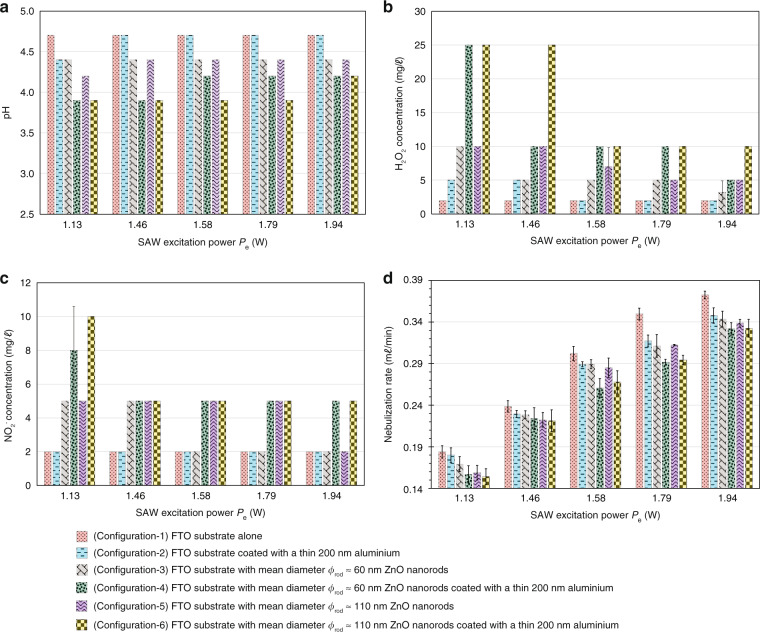
Characterization of plasma-activated aerosols generated via different configurations. **a** pH, **b** H_2_O_2_ concentration and **c** NO${}_{2}^{-}$ concentration of the collected plasma-activated aerosols generated at different SAW excitation powers (*P*_e_ = 1.13, 1.46, 1.58, 1.79, 1.94 W) for the six different configurations used to plasma-activate the purified water feed to the SAW nebulizer (see legend). For the cases where ZnO nanorods were coated on the FTO substrates, two different mean diameters of the nanorods were used: *ϕ*_rod_ ≈ 60 and 110 nm (Fig. 2). Prior to plasma treatment, the water from the reservoir (Fig. 1a) had a baseline pH of 5, and negligible (undetectable) H_2_O_2_ and NO${}_{2}^{-}$ concentrations. The height and angle between the FTO substrate and the ground electrode (aluminium tape) were fixed at *H*_pls_ = 16.6 mm and *θ*_FTO_ = 90^∘^, respectively (Fig. 1b), and the applied voltage across the FTO substrate and ground electrode was held at *V*_p_ = 15 kV. **d** Measured nebulization rate *Q*_A_ as a function of the SAW excitation power *P*_e_ for all six configurations. Mean values and standard deviations were calculated from ten replicate experiments

We quantified the pH and the H_2_O_2_ and NO${}_{2}^{-}$ concentrations of the collected aerosols under different nebulization rates (achieved by adjusting the input SAW excitation power *P*_e_) for each of the six aforementioned configurations, noting a pH value of 5 for purified water in the reservoir prior to plasma treatment and negligible (undetectable) baseline H_2_O_2_ and NO${}_{2}^{-}$ concentrations. Among the six configurations, it was observed from Fig. 3 that the pH value is at its highest (4.7) and the H_2_O_2_ and NO${}_{2}^{-}$ concentrations at their lowest (2 mg/*ℓ*) with the bare and aluminium-coated FTO substrates (Configuration-1 and Configuration-2, respectively) due to the relatively weaker plasma intensity in the absence of the nanorods, thereby resulting in the generation of fewer reactive species in the plasma-activated aerosols. A comparison between Configuration-2 with Configuration-4 and Configuration-6 further shows that the presence of the nanorods leads to significantly higher H_2_O_2_ concentration. We note, however, that the pH decreases to 4.4, and the H_2_O_2_ concentration increases to 5 mg/*ℓ* at lower SAW excitation power *P*_e_ and hence lower nebulization rates. In brief, this is likely because of the increased exposure duration of the water to the plasma as a result of the slower transport of water through the paper strip, which is typically equal to the nebulization rate, although we will subsequently discuss the interdependency of the reactive species generation and the nebulization rate below.

There is a notable improvement in the plasma activation of the aerosols when the ZnO nanorods are present (Configuration-3 and Configuration-5), albeit only marginally, with the pH values decreasing slightly to around 4.4 and the H_2_O_2_ concentration increasing slightly to ~5 mg/*ℓ*; no appreciable change was noted in the NO${}_{2}^{-}$ concentration (~2 mg/*ℓ*). This is because of the high electrical resistivity of the ZnO nanorods. Thicker nanorods (Configuration-5: *ϕ*_rod_ ≈ 110 nm) are observed to lead to slightly higher concentration of reactive species compared to their thinner counterparts (Configuration-3: *ϕ*_rod_ ≈ 60 nm), likely because the nanorods are more densely packed, as can be seen from Fig. 2(a–ii) and (b–ii), which result in more uniform plasma generation and exposure along the paper strip. Further marginal improvements in the plasma activation can be achieved by lowering the SAW excitation power *P*_e_ and hence the nebulization rate to prolong exposure to the plasma of the purified water being transported through the paper strip, as previously observed with the substrates devoid of the nanorods (Configuration-1 and Configuration-2).

Increasing the electrical conductivity of the plasma generator by coating the ZnO nanorods with a thin aluminium layer (Configuration-4 and Configuration-6), on the other hand, provides the most substantial improvement in plasma activation. Taken in tandem, the aluminium coating together with the thick ZnO nanorods (Configuration-6) at the lowest SAW excitation power *P*_e_ of 1.13 W and hence nebulization rate (As seen from Table 1, Configuration-6 provides the most efficient means of plasma activation, decreasing the pH to 3.9 and increasing the H_2_O_2_ concentration by around one order of magnitude to 25 mg/*ℓ* and the NO${}_{2}^{-}$ concentration by up to fivefold to 10 mg/*ℓ*).

**Table 1:** pH, H_2_O_2_ concentration and NO${}_{2}^{-}$ concentration of the collected plasma-activated aerosols as a function of the nebulization rate *Q*_A_, which was altered by varying the SAW excitation power *P*_e_, for Configuration-6 (Fig. 3) with a height of *H*_pls_ = 15 mm between the aluminium-coated nanorods and the ground electrode (the smallest electrode separation gap that ensures stable plasma generation). The angle between the aluminium/nanorod coated FTO substrate relative to the ground electrode is fixed at *θ*_FTO_ = 90^∘^, and the voltage applied across the nanorods and the ground electrode is *V*_p_ = 15 kV. Mean values and standard deviations were calculated from ten replicate experiments

	Nebulization rate *Q*_A_ (m*ℓ*/min)				
	0.12	0.17	0.23	0.28	0.32
	SAW excitation power *P*_e_ (W)				
	1.13	1.46	1.58	1.79	1.94
pH	3.0 ± 0.0	3.0 ± 0.0	3.0 ± 0.0	3.3 ± 0.0	3.3 ± 0.0
H_2_O_2_ concentration (mg/*ℓ*)	30 ± 0	28 ± 3	25 ± 0	16 ± 8	10 ± 0
NO${}_{2}^{-}$ concentration (mg/*ℓ*)	10 ± 0	10 ± 0	7 ± 3	5 ± 0	5 ± 0

**Fig. 4:**
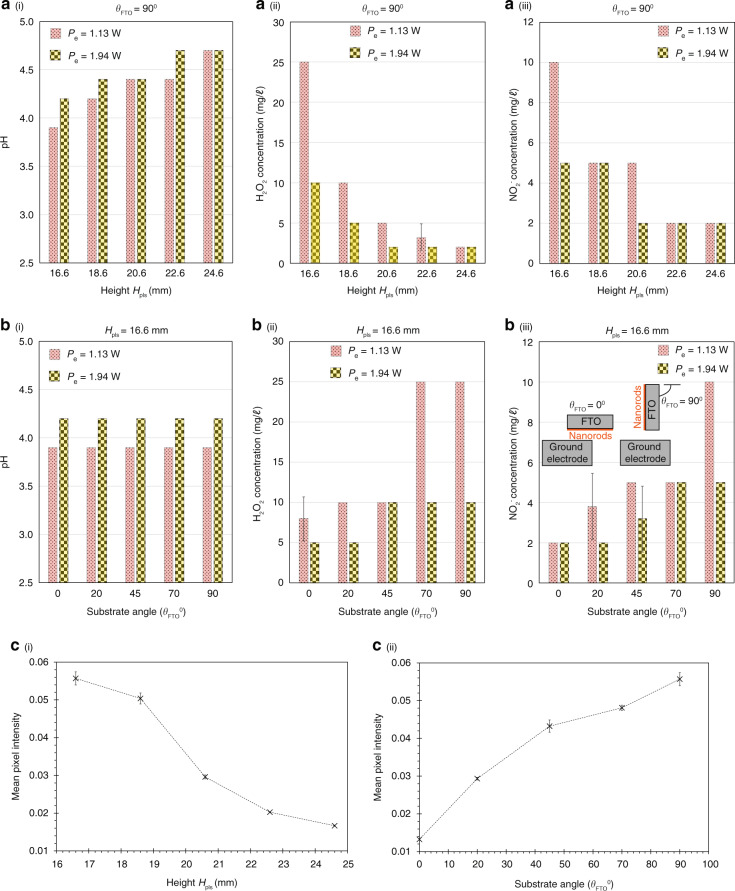
Characterization of plasma-activated aerosols generated via different substrate angles and heights. Effect of varying (**a**) the height *H*_pls_, and, **b** the angle *θ*_FTO_ (see the definition of the angle in the inset of (**b**–iii)) between the aluminium-coated ZnO nanorods on the FTO substrate (Configuration-6) in Fig. 3) and the ground electrode (aluminium tape; see Fig. 1b) on (i) the pH, (ii) the H_2_O_2_ concentration, and, (iii) the NO${}_{2}^{-}$ concentration of the collected plasma-activated aerosols generated at two SAW excitation powers *P*_e_ = 1.13 and 1.94 W. In **a**, the substrate angle was maintained at *θ*_FTO_ = 90^∘^, whereas in **b**, the height was maintained at *H*_pls_ = 16.6 mm. In both **a** and **b**, the voltage applied across the nanorods and the ground electrode was held at *V*_p_ = 15 kV. Mean values and standard deviations were calculated from ten replicate experiments. **c** The strengths of the plasma generated at different (i) heights *H*_pls_ and (ii) angles *θ*_FTO_ were quantified via the mean pixel intensity analysis, that is, for each parameter, five images (4130 pixles × 3284 pixels) of the plasma were captured using a camera (Nikon D5600) equipped with a magnification lens (Nikon, AF-S Micro Nikkor 105mm 1:2.8G ED) and the mean pixel intensities were calculated using the Mathematica software (Wolfram Research, Champaign, IL, USA). Higher mean pixel intensity values represent stronger plasma

The most significant increase in the efficiency of plasma activation, nevertheless, can be achieved by utilizing smaller gaps between the FTO substrate and the ground electrode (Fig. 4a, c–i), ensuring that the overall nanorod and substrate orientation is orthogonally aligned to the ground electrode and paper strip (that is, *θ*_FTO_ = 90^∘^; Fig. 4b, c–ii). The former (that is, smaller *H*_pls_) is expected since this results in a larger electric field for a given applied voltage across the nanorod and ground electrode *V*_p_, although we note that a minimum separation value *H*_pls_ = 15 mm is necessary to ensure stable plasma generation (*H*_pls_ = 16.6 mm in the case when the nanorods are absent, that is, Configuration-1 and Configuration-2). The latter (that is, mounting the substrate and hence the nanorods orthogonally to the ground electrode *θ*_FTO_ = 90^∘^, as opposed to in parallel *θ*_FTO_ = 0^∘^), appears to be counterintuitive, although we note that the electric field is typically more intense at the singular tip of a point electrode configuration compared to that for a parallel plate electrode configuration, therefore leading to greater dielectric breakdown and hence more intense plasma generation around the substrate tip that resembles a singular point.

It is worth noting the nuanced interrelationship between the concentration of the reactive species and the nebulization rate. While we have observed that lower SAW excitation powers *P*_e_ and hence nebulization rates give rise to more efficient plasma activation and hence the generation of reactive species due to longer exposure durations of the water in the paper strip to the plasma, the resultant increase in the reactive concentration species results in an enhancement in the electrical conductivity of the water sample that concomitantly reduces the contact angle of the meniscus front and hence the thickness of the film emanating from the paper strip where nebulization occurs^15^. As seen in Tables 2 and 3, this, in turn, results in a decrease in the aerosol size *ϕ*_d_ and the nebulization rate *Q*_A_, which have been previously shown to decrease with the film thickness as ${\phi }_{{{{\rm{d}}}}} \sim {H}_{{{{\rm{f}}}}}^{8/3}$ and ${Q}_{{{{\rm{A}}}}} \sim {H}_{{{{\rm{f}}}}}^{2}$, respectively^15,20,46^.

**Table 2:** Measured aerosol diameter *ϕ*_d_ and atomization rate *Q*_A_ for different values of pH and electric conductivity *σ*_e_ of the collected plasma-activated aerosols. The plasma was generated using the FTO substrate with the aluminium-coated ZnO nanorods (Configuration-6 in Fig. 3) mounted at *θ*_FTO_ = 90^∘^. The distance between the FTO substrate and the grounded electrode *H*_pls_ was varied to obtain the different pH values (the solution with pH = 5 represents that of purified water). Both LN substrates without and with an aluminium coating in the nebulization region (Fig. 1(c)) were employed, and the SAW excitation power *P*_e_ was held constant at 1.13 W

		Nebulization rate *Q*_A_ (m*ℓ*/min)		Aerosol diameter *ϕ*_d_ (*μ*m)	
	Electrical	LN substrate	LN substrate	LN substrate	LN substrate
	conductivity	without	with	without	with
pH	*σ*_e_ (*μ*S/cm)	Al coating	Al coating	Al coating	Al coating
5.0	12.6	0.185 ± 0.006	0.192 ± 0.005	11 ± 2	11 ± 2
4.2	113.1	0.163 ± 0.005	0.198 ± 0.004	9 ± 2	10 ± 2
3.6	258.4	0.134 ± 0.007	0.175 ± 0.006	8 ± 2	10 ± 2
3.0	338.0	0.123 ± 0.009	0.167 ± 0.005	7 ± 1	10 ± 2

As such, a balance exists between reducing the nebulization rate to enhance the plasma activation of the water sample and the need to increase the efficiency of aerosol delivery for effective surface treatment. To address this, that is, increase the concentration of reactive species generated whilst maintaining the nebulization rate, we suppress the electric field generated on the piezoelectric LN substrate due to the propagation of the SAW on it (the SAW is a coupled electromechanical wave^26,47^) while leaving the mechanical component of the SAW intact by sputter depositing a thin (200 nm) aluminium layer in the nebulization region on the substrate, as shown in Fig. 1(c–ii). From Table 3, we observe the effect of this feature to lead to a thicker film and hence an enhancement in the nebulization rate by ~36%.

**Table 3:** Measured mean length *L*_f_ and height *H*_f_ of the plasma-activated water film emanating from the leading edge of the paper strip driven by the SAW (see inset (a–iv) in Fig. 1) on LN substrates without and with an aluminium coating in the nebulization region (Fig. 1(c)). The solution with pH=5 represents that of purified water.

	Uncoated LN substrate		Al-coated LN substrate	
Plasma-activated	Length *L*_f_	Height *H*_f_	Length *L*_f_	Height *H*_f_
water	(*μ*m)	(*μ*m)	(*μ*m)	(*μ*m)
pH 5.0	484	207	546	226
pH 3.3	429	156	500	199
Δ%	12%	27%	9%	12%

**Fig. 5:**
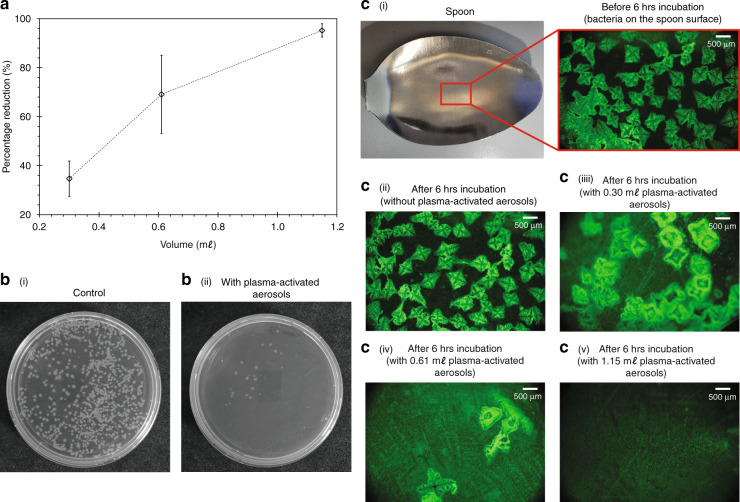
Plasma-activated aerosols for bacteria inactivation. **a** Efficiency of the plasma-activated aerosols for bacteria inactivation based on the estimated percentage reduction in bacterial colony count. The plasma-activated aerosols were directly sprayed with the SAW nebulizer onto the agar plate containing the bacteria suspension. The standard deviation in *η*_e_ for each volume was independently calculated from triplicate experiments. Representative images showing **b**–i the presence of *Escherichia coli* (E. coli) colonies on the agar plate for the control group (without the application of plasma-activated aerosols), and, **b**–ii the inactivation of E. coli on the agar plate following direct spray deposition of 1.15 m*ℓ* of plasma-activated aerosols onto the plate over a nebulization time of approximately 9.5 min. Images showing **c**–i an area on the stainless steel spoon surface coated with fluorescently labelled E. coli colonies before the 6 h incubation period; after 6 h incubation period, **c**–ii the presence of E. coli colonies without plasma-activated aerosols, reduction of E. coli colonies following spray deposition of **c**–iii 0.30 m*ℓ*, **c**–iv 0.61 m*ℓ*, and **c**–v 1.15 m*ℓ*, plasma-activated aerosols; lower fluorescence intensities indicate the bacteria have been inactivated as the green fluorescent proteins have been leaked due to the lost of membrane integrity. For both demonstrates of the bacterial inactivation on the agar plates in **a** and **b** and stainless steel spoon in **c**, the plasma-activated aerosols were produced using Configuration-6, with *H*_pls_ = 15 mm, *θ*_FTO_ = 90^∘^, and a SAW excitation power of *P*_e_ = 1.13 W (Table 1).

Compared to the preceding state-of-the-art that comprises a two cometary plasma setup^15^, the present system is not only able to deliver improvements of up to 20% and 127% in the aerosol H_2_O_2_ and NO${}_{2}^{-}$ concentrations, respectively, at comparable nebulization rates, but also doing so in a compact and miniaturizable setup that facilitates ease of integration into a portable device that can be easily deployed for field use. Such an enhancement also translates into a reduction in the required nebulization time to achieve the desired bacteria inactivation efficiency. For Configuration-6 (Table 1), we observe from Fig. 5a, b the percentage reduction in the bacterial colony count *η*_e_ to increase as larger quantities of the plasma-activated aerosols are directly sprayed onto the *Escherichia coli* (E. coli) suspension in the agar plates. For example, a percentage reduction of up to *η*_e_ = 95% can be achieved by spraying 1.15 m*ℓ* of plasma-activated aerosol (pH 3.0, 30 mg/*ℓ* H_2_O_2_), which is comparable to the volumes administered in the preceding study with the two cometary plasma setup (1.18 m*ℓ*; pH 3.0, 25 mg/*ℓ* H_2_O_2_)^15^, but over significantly reduced nebulization times (approximately 9.5 min, as opposed to 30 min in Ref. ^15^). As can be seen in Fig. 5c, simultaneous spraying the plasma-activated aerosols onto a stainless steel spoon coated with bacteria to mimic a more realistic surface shows efficient inactivation of the pathogens with just 1.15 m*ℓ* of the plasma-activated aerosols.

## Conclusion

We have demonstrated in this work the coupling of a nanoscale plasma generator with a nanoelectromechanical (SAW) nebulizer for efficient direct spraying of plasma-activated aerosols onto surfaces for pathogen inactivation and decontamination. The nanoscale plasma generator comprises a ZnO nanorod carpet which essentially resembles an ensemble of nanoneedles acting as near-singular point charge sources, which when raised to a high electric potential, is highly efficient in breaking down the air around it to generate atmospheric pressure plasma. In addition to comprising a significantly more compact setup compared to the cometary plasma generator utilizing macroscopic charging sources that were used in preceding work^15^, thereby facilitating ease of integration with the chips-cale SAW nebulizer to constitute a lightweight, portable system that enables field use, we show that the combined nanoscale plasma and aerosol generator setup is capable of efficiently producing plasma-activated aerosols in situ with considerably higher reactive species concentrations, namely 20% and 127% increases in the aerosol H_2_O_2_ and NO${}_{2}^{-}$ concentrations, respectively. This, in turn, translates into a significant reduction in the requisite administration (nebulization) time from 30 min with the predecessor cometary plasma discharge device to 9.5 min with the current setup.

## Materials and methods

### Fabrication of ZnO nanorods

Fluorine-doped tin oxide (FTO) glass substrates (735167-1EA, Sigma-Aldrich (M) Sdn. Bhd., Selangor, Malaysia) with length *L*_FTO_ = 1 cm, height *H*_FTO_ = 1 cm and width *W*_FTO_ = 2.3 mm were first cleaned with acetone using cotton buds followed by a delicate task wipe (Kimtech^®^ Science Kimwipes™; Kimberly–Clark Worldwide Inc., Irving, TX, USA) before being fully submerged in glass jars filled with acetone and sonicated in an ultrasonic cleaner (Thermo-10D; Thermoline Scientific Equipment Pty. Ltd., Wetherill Park, NSW, Australia) for 15 min. The acetone is then replaced with isopropanol (IPA) and the sonication process repeated for another 15 min. The FTO substrates were subsequently placed in a glass petri dish and dried using a hotplate (Corning^®^ PC-420D; Corning Inc., New York, USA) for 15 min at 110 ^∘^C. After the FTO substrates were fully dried, they were placed facing upwards (identified using a multimeter) in a UV ozone cleaner (E511; Ossila Ltd., Sheffield, UK) with a power rating of 120 W for 10 min to remove any remaining organic contaminants on the substrate surface^48^.

Following the cleaning processes, a ZnO seed layer was deposited in a small circular region onto the FTO substrates (Fig. 2c) by means of a sol-gel method; the circular pattern was achieved by masking the substrate with adhesive tape during deposition. Zinc acetate dihydrate (Zn(CH_3_CO_2_)_2_ ⋅ 2H_2_O; ZnAc) was used as the starting material to prepare the seed layer along with ethanol. 0.3 M ZnAc (383058; Sigma-Aldrich Inc., St. Louis, MO, USA) was measured using a precision balance (FX-300i; A&D Australasia Pty. Ltd., Thebarton, SA, Australia) and placed in a vial with a clean spatula. Ten milliliter absolute ethanol was then measured with a micropipette (Pipetman^®^ M P10mL; Gilson Inc., Middleton, WI, USA) and dispensed into the same vial. The vial was then sonicated in an ultrasonic cleaner for 5 min to ensure the ZnAc was fully dissolved in the ethanol and the solution spin coated onto the FTO substrate. A 25 *μℓ* of the solution was measured with the micropipette and deposited onto the substrate placed in a vacuum spin coater (VTC-100; MTI Corp. Richmond, CA, USA) and spun at 3000 rpm for 30 s. The spun coated FTO substrates were subsequently placed onto a glass petri dish and dried using the hotplate at 110 ^∘^C for 10 min and subsequently at 50 ^∘^C for 15 min. To obtain a uniform layer, the aforementioned process was repeated and the solution spun coated onto the FTO substrates for a further two times. Following completion of the three spin coating cycles, the substrates were placed onto a ceramic holder and wrapped with a layer of aluminium foil with small holes. The ceramic holder was then placed in the middle of a high-temperature tube furnace (RHTC 80-710; Nabertherm Inc., Bahnhofstr, Lilienthal, Germany) heated to 400 ^∘^C over 30 min and annealed for 3 h to obtain a stable film with crystallization centres^49^. We note that longer annealing times can result in an increase in nanorod diameters^43^.

### Generation of plasma and plasma-activated water

The aluminium-coated ZnO nanorods grown on the FTO substrate was used to generate atmospheric pressure plasma by connecting the positive terminal of a high voltage DC power supply (SRS PS375; FuG Elektronik GmbH, Rosenheim, Germany) to the nanorods and connecting the negative terminal to aluminium tape that constituted the ground electrode. Under a sufficiently high electric field (>10 kV) between the terminals, a stable atmospheric pressure plasma was generated on the nanorods, as illustrated in Fig. 1a, b. We note that the paper strip through which water was transported from the reservoir to the SAW device was placed atop the aluminium tape such that the entire surface of the paper strip was exposed to the plasma (Fig. 1(b–i)), in order to generate the plasma-activated water that is to be subsequently aerosolized by the SAW nebulizer.

### Generation of plasma-activated aerosols

The SAW nebulization device consisted of a 128^∘^-rotated Y-cut X-propagating single-crystal lithium niobate (LN) piezoelectric substrate (Roditi Ltd., London, UK) on which a focusing elliptical single-phase unidirectional transducer (FE-SPUDT) was fabricated using standard UV photolithography. The resonant frequency of the device, in this case, *f*_SAW_ = 30.5 MHz, is determined by the spacing and width of the fingers in the FE-SPUDT. The resonant frequency of the SAW device is determined by *f*_SAW_ = *c*_SAW_/*λ*_SAW_, where *c*_SAW_ ≈ 3965 m/s is the speed of the SAW travelling along the substrate surface. With *f*_SAW_ = 30.5 MHz, the SAW wavelength is approximately *λ*_SAW_ ≈ 130 *μ*m. The SAW is launched by applying a sinusoidal electrical signal at the resonant frequency *f*_SAW_, generated with a function generator (AFG1062; Tektronix Inc., Beaverton, OR, USA) and amplifier (ZHL-5W-1; Mini-Circuits Inc., NY, USA), to the FE-SPUDT. To increase the efficiency of operation, we amplitude modulate the signal with a modulation frequency *f*_m_ = 1 kHz in order to reduce the power required to maintain continuous nebulization^20^. The total power of the electric signal to the FE-SPUDT can be determined from *P*_e_ = *P*_c_(1 + *m*^2^/2), where *P*_c_ = *V*_c_*I*_c_ is the RMS power of the carrier signal and *m* = *V*_m_/*V*_c_ is the modulation index^50^, whereby ${V}_{{{{\rm{m}}}}}=\left({V}_{\max }-{V}_{\min }\right)/2$ is the modulated signal voltage and ${I}_{{{{\rm{c}}}}}=({I}_{\max }+{I}_{\min })/2$ is the carrier signal current. ${V}_{\max }$ and ${V}_{\min }$ are the maximum and minimum RMS voltages, whereas ${I}_{\max }$ and ${I}_{\min }$ are the maximum and minimum RMS currents, respectively, measured using an oscilloscope (TDS 2012C; Tektronix Inc., Beaverton, OR, USA) connected with voltage (TPP 0201; Tektronix Inc., Beaverton, Oregon, USA) and current (P6022; Tektronix Inc., Beaverton, OR, USA) probes.

A paper strip (WIP-100DLE; Suorec Sdn. Bhd., Batu Berendam, Melaka, Malaysia) of width *W*_ps_ ≈ 8 mm, length *L*_ps_ ≈ 80 mm and thickness *H*_ps_ ≈ 300 *μ*m was used as a porous conduit to transport water from the reservoir to the LN substrate (Fig. 1). More specifically, the SAW generates a negative pressure in the paper strip such that the water is drawn from the reservoir and wicks through the paper strip, from which it emanates to form a thin film on the LN substrate where it is nebulized to form aerosol droplets with diameters on the order *ϕ*_d_ ~ 1 *μ*m. When the water in the paper strip is exposed to the plasma generated on the aluminium-coated ZnO nanorods above it, the water is plasma-activated such that the plasma-activated aerosols are produced downstream via nebulization by the SAW device. The increased conductivity of plasma-activated water samples has been observed to result in a decrease in the liquid film thickness on the LN substrate compared to that for pure water samples^15^. This, in turn, resulted in smaller aerosol droplet sizes^16^ and nebulization rates. To compensate for the nebulization rate, we sputter deposited (MIMOS Semiconductor (M) Sdn. Bhd., Kuala Lumpur, Malaysia) a thin 200 nm conductive (aluminium) layer in the nebulization zone where the thin film is formed on the SAW device ahead of the FE-SPUDT.

### Quantification of the nebulization rate

The rate at which the plasma-activated aerosols was nebulized was quantified based on the weight of aerosols collected over a 5 min duration. Briefly, the aerosols were collected in a test tube (Pyrex^®^ TE-32; Iwaki Glass, Sumedang, West Java, Indonesia), which was positioned at the location near where the aerosols were generated. The weight of the empty test tube (before the experiment) *m*_i_ and that of the test tube containing the collected aerosols (after 5 min) *m*_a_ were measured on a weighing scale (MS303S/01; Mettler Toledo, Greifensee, Switzerland). The nebulization rate *Q*_A_ can then be estimated from ${Q}_{{{{\rm{A}}}}}=\left[({m}_{{{{\rm{i}}}}}-{m}_{{{{\rm{a}}}}})/5\,{{{\rm{mins}}}}\right]\times 1\,{{{\rm{m}}}}\ell /$g.

### Characterization of plasma-activated aerosols

To quantify the effectiveness of the plasma-activated aerosols, we measured the concentration of nitrite ions (NO${}_{2}^{-}$) and hydrogen peroxide (H_2_O_2_) together with the solution pH of the aerosols collected as they deposit into a test tube (Pyrex^®^ TE-32; Iwaki Glass, Sumedang, West Java, Indonesia). pH-indicator strips (Supelco MQuant^®^ pH 2.5-4.5 & Supelco MQuant^®^ pH 4.0–7; Merck KGaA, Darmstadt, Germany) were used to measure the pH of the collected plasma-activated aerosols. The H_2_O_2_ concentration was measured using a colorimetric test (Supelco MQuant^®^ 1.10011.0001; Merck KGaA, Darmstadt, Germany) and peroxide test strips (Quantofix Peroxide 100-91312; Macherey-Nagel GmbH, Düren, Germany), whereas the NO${}_{2}^{-}$ ion concentration was measured using nitrite test strips (Supelco MQuant^®^ 1.10007.0001; Merck KGaA, Darmstadt, Germany). The effect of varying the nebulization rate *Q*_A_ (by varying the input power to the SAW device *P*_e_), the nanorod diameter *ϕ*_rod_, the FTO substrate angle *θ*_FTO_, and the DC electric field across the nanorods (by varying the separation between the nanorods and the ground aluminium tape *H*_pls_) were investigated. Aerosol diameters *ϕ*_d_ were estimated by examining aerosols collected on a slide under a microscope (Eclipse Ci-E; Nikon Inc., Minato, Tokyo, Japan), whereas the length *L*_f_ and height *H*_f_ of the thin film emanating from the paper strip where nebulization ensues were estimated using a high-speed camera (Phantom M310; Vision Research Inc., Wayne, NJ, USA).

### Quantification of bacterial inactivation

The bacteria inactivation efficiency of the plasma-activated aerosols was analysed by spraying them directly onto bacterial colonies plated on agar plates. Briefly, 500 *μℓ* E. coli (BL21(DE3) strains) was cultured in 50 m*ℓ* of Luria-Bertani (LB) broth for 12 h at 37 ^∘^C and 200 rpm to reach a logarithmic growth phase to approximately 10^8^ CFU/m*ℓ*. Next, 100 *μℓ* aliquots from the culture was centrifuged at 5000 rpm for a duration of 5 min and resuspended in 1 m*ℓ* of 0.9% sodium chloride solution. The suspension was then serially diluted until it achieved a concentration of ~10^3^ CFU/m*ℓ,* and 50 *μℓ* of diluted bacteria suspension was plated onto LB agar plates. Subsequently, the surface of the plated LB agar plates was directly exposed to various volumes—0.38, 0.61 and 1.15 m*ℓ*—of plasma-activated aerosols that had been produced via the hybrid system, which consists of the plasma generator and the SAW nebulizer. After the bacteria colonies were exposed to the plasma-activated aerosols, the LB agar plates were incubated at 37 ^∘^C for about 12 h. The bacteria inactivation efficiency of the plasma-activated aerosol was then quantified based on the percentage reduction of CFUs grown between treated and untreated (control) agar plates, which can be estimated from

1 $${\eta }_{{{{\rm{e}}}}}=\frac{{{{{\rm{CFU}}}}}_{{{{\rm{control}}}}}-{{{{\rm{CFU}}}}}_{{{{\rm{treated}}}}}}{{{{{\rm{CFU}}}}}_{{{{\rm{control}}}}}}\times 100 \% .$$

### Bacterial inactivation on a stainless steel surface

To demonstrate the possibility of disinfection on a more realistic surface, the plasma-activated aerosols were sprayed directly onto a stainless steel spoon pre-coated with E. coli. Prior to the experiment, the E. coli BL21(DE3) strains were cultured in Luria-Bertani (LB) broth at 37 ^∘^C and 200 rpm until the optical density of the culture reached 0.8. This was followed by the addition of 0.5 mM of isopropythio-*β*-D-galactosidase (IPTG; Sigma-Aldrich, St. Louis, MO, USA) to the cell culture for induction of green fluorescent protein expression, followed by incubation for 14 h at 30 ^∘^C and 200 rpm. Next, a 100 *μℓ* aliquot bacterial suspension was centrifuged at 5000 rpm for 5 min and the pellet was resuspended in 1 m*ℓ* of 0.9% (w/v) sodium chloride solution. The suspension was then serially diluted until the cell concentration reached approximately 10^3^ CFU/m*ℓ*. Finally, 50 *μℓ* of the diluted bacteria suspension was coated on four spoons, one of which was used as the control (without any treatment), whereas the other three were treated with 0.30, 0.61 and 1.15 m*ℓ* of the plasma-activated aerosols. All spoons were incubated at 37 ^∘^C for 6 h prior to their inspection under a fluorescence microscope (BX41M, Olympus, Tokyo, Japan).
